# Distinct Actions of the Thyroid Hormone Transporters Mct8 and Oatp1c1 in Murine Adult Hippocampal Neurogenesis

**DOI:** 10.3390/cells11030524

**Published:** 2022-02-02

**Authors:** Steffen Mayerl, Andrea Alcaide Martin, Reinhard Bauer, Markus Schwaninger, Heike Heuer, Charles ffrench-Constant

**Affiliations:** 1 Department of Endocrinology, Diabetes & Metabolism, University Hospital Essen, University of Duisburg-Essen, 45147 Essen, Germany; andrea.alcaidemartin@uk-essen.de (A.A.M.); heike.heuer@uni-due.de (H.H.); 2 MRC Centre for Regenerative Medicine, University of Edinburgh, Edinburgh EH16 4UU, UK; cffc@uea.ac.uk; 3 Institute of Molecular Cell Biology, Jena University Hospital, 07745 Jena, Germany; reinhard.bauer@med.uni-jena.de; 4 Institute for Experimental and Clinical Pharmacology and Toxicology, University of Lübeck, 23562 Lübeck, Germany; markus.schwaninger@uni-luebeck.de; 5 Faculty of Medicine and Health Sciences, University of East Anglia, Norwich NR4 7TJ, UK

**Keywords:** adult hippocampal neurogenesis, Allan-Herndon-Dudley Syndrome, Mct8, Oatp1c1, thyroid hormone, Slc16a2, Slco1c1

## Abstract

Inactivating mutations in the thyroid hormone (TH) transporter monocarboxylate transporter 8 (MCT8) result in Allan-Herndon-Dudley Syndrome, a severe form of psychomotor retardation, while inactivating mutations in another TH transporter, organic anion transporting polypeptide 1c1 (OATP1C1), are linked to juvenile neurodegeneration. These diseases point to essential roles for TH transporters in CNS function. We recently defined the presence of Mct8 in adult hippocampal progenitors and mature granule cell neurons and unraveled cell-autonomous and indirect requirements for Mct8 in adult hippocampal neurogenesis. Here, we investigated whether Oatp1c1 is involved in the hippocampal neurogenic process in concert with Mct8. We detected Oatp1c1 gene expression activity and transcripts in subsets of progenitors, neurons and niche cells in the dentate gyrus. Absence of Oatp1c1 resulted in increased neuroblast and reduced immature neuron numbers in 6-month-old Oatp1c1ko and Mct8/Oatp1c1 double knockout (M/Odko) mice. Reduced EdU-label retention in Mct8ko and M/Odko mice confirmed the impact of Mct8 on neuron formation. In contrast, no significant effect of Oatp1c1 loss on granule cell neuron production and anxiety-like behavior in the open field arena were seen. Together, our results reinforce that distinct actions of each TH transporter are required at multiple stages to ensure proper adult hippocampal neurogenesis.

## 1. Introduction

Thyroid hormones (THs) are critical regulators of CNS development and function [1,2,3]. To enable these CNS effects, TH transporters facilitate TH uptake across the blood-brain barrier (BBB) and blood-cerebrospinal fluid barrier (BCSFB) and also mediate TH transport into neural target cells [4]. One of these, the monocarboxylate transporter 8 (MCT8) encoded by the *SLC16A2* gene, exhibits the highest specificity towards the prohormone 3,3′,5,5′-tetraiodothyronine (thyroxine; T4) and the receptor active form 3,3′,5-triiodothyronine (T3) [5]. Inactivating mutations in Mct8 cause Allan-Herndon-Dudley Syndrome (AHDS), a severe form of psychomotor retardation that is accompanied by abnormal serum TH values and symptoms of peripheral thyrotoxicosis [6,7,8]. In addition, AHDS patients demonstrate hallmarks of hypothyroid brain development, presumably due to an insufficient TH transport into the CNS. Inactivating mutations in the *SLCO1C1* gene encoding for another transporter, the T4 transporter organic anion transporting polypeptide 1c1 (OATP1C1), are linked to a juvenile form of neurodegeneration, suggesting that OATP1C1 also plays a distinct role in TH transport in the human CNS [9]. 

In studies using transgenic knockout mice to model human diseases caused by TH transporter deficiencies, Mct8 knockout (Mct8ko) mice replicate the peripheral alterations in TH homeostasis, but present only slightly reduced brain TH concentrations and no apparent gross neurological or motor abnormalities [10,11]. Only a concomitant ablation of Mct8 and Oatp1c1 results in profoundly diminished TH passage into the CNS and AHDS-like symptoms in mice [12]. The requirement of a double knockout for the AHDS phenotype is explained by a species-specific expression pattern of Oatp1c1 in rodent but not primate blood brain barrier cells [13,14,15]. Asking whether the loss of transport across the BBB is solely responsible for the AHDS-like phenotype, we recently used a conditional knockout strategy to examine the role of Mct8 in hippocampal neurogenesis, a key process for proper learning and memory function as well as mood control known to be highly dependent on proper TH signaling [16]. We showed that cell-autonomous Mct8 expression in neuroblasts of the adult hippocampal lineage is crucial for proper differentiation into immature neurons and the formation of new granule cell neurons [17]. However, a key question not yet answered is whether, as suggested by the human neurodegenerative phenotype, Oatp1c1 also has a cell-autonomous effect on hippocampal neurogenesis that contributes to the AHDS-like phenotype of the Mct8/Oatp1c1 double knockout (M/Odko) in addition to any effects exerted by the loss of BBB transport function. 

Here, we address this question by defining Oatp1c1 expression in the hippocampal dentate gyrus and comparing adult hippocampal neurogenesis at two different postnatal time points in Oatp1c1 knockout (Oatp1c1ko) with control, Mct8ko and M/Odko mice. 

## 2. Materials and Methods

### 2.1. Study Approval

All animal studies were executed in accordance with the European Union (EU) directive 2010/63/EU and approved by the Animal Welfare Committees of the Landesamt für Natur, Umwelt und Verbraucherschutz Nordrhein-Westfalen (LANUV; Recklinghausen, Germany) and of the Thüringer Landesamt für Lebensmittelsicherheit und Verbraucherschutz (TLLV; Bad Langensalza, Germany) as well as in compliance with UK Home Office regulations and local guidelines by The University of Edinburgh to reduce the number of animals used and the severity of any procedures.

### 2.2. Animals

Mct8ko, Oatp1c1ko, and M/Odko mice have been generated and genotyped as described before [10,12,18]. Transgenic animals expressing a Tamoxifen inducible Cre recombinase driven by the endogenous Oatp1c1 (gene name *Slco1c1*) locus [19] as well as harboring a reporter allele consisting of a loxP-flanked STOP cassette that prevents transcription of a CAG promotor driven EYFP construct (Gt(ROSA)26Sor^tm3(CAG-EYFP)Hze^) [20] were bred and genotyped as before. All mice were bred on a C57BL/6 background.

Mice were kept at constant temperature (22 °C) on a 12 h light, 12 h dark cycle and were provided with standard laboratory chow and water ad libitum. Male mice positive for the Oatp1c1-CreERT2 and EYFP transgenes at the age of 2 or 6 months were i.p. injected with 40 µg/g body weight/day tamoxifen (10 mg/mL; Thermo Fisher Scientific, Waltham, MA, USA) for five consecutive days. Animals were deeply anaesthetized by ketamine/xylazine application and subjected to terminal perfusion fixation 72 h after the last tamoxifen application. For EdU label retention studies, 2- or 6-month-old mice were i.p. injected once with 100 µL EdU (10 mg/mL; Thermo Fisher Scientific, Waltham, MA, USA) 3 or 28 days prior to organ collection. After perfusion fixation with 4% paraformaldehyde/PBS, brains were cryo-protected with 30% sucrose, snap-frozen in isopentane on dry ice and kept at −80 °C. FISH experiments were performed utilizing brains from P180 Wt males that were snap-frozen in isopentane on dry ice and stored at −80 °C. Behavioral assessments were conducted with female mice at the age of 1 year. 

### 2.3. Immunofluorescence Studies

Coronal forebrain cryosections (16 µm) containing the hippocampus were thaw-mounted on superfrost slides (Thermo Fischer Scientific, Waltham, MA, USA), post-fixed with 4% PFA for 10 min and permeabilized with 0.1% Triton X-100/0.1 M glycine. For EdU lineage tracing, an EdU click-it reaction was carried out following the manufacturer’s instructions using the Click-iT^®^ EdU Alexa Fluor^®^ 647 Imaging Kit (Thermo Fisher Scientific, Waltham, MA, USA). Sections were treated with a blocking buffer (PBS containing 10% goat serum and 0.2% Triton X-100) containing 1:40 mouse-on-mouse blocking (MOM) reagent (Vector Laboratories, Burlingame, CA, USA). Subsequently, sections were incubated with primary antibodies in the blocking buffer overnight at 4 °C. Following washing with PBS, sections were incubated with fluorescent Alexa Fluor (AxF) 488, 555, 568 or 647 labelled secondary antibodies (all 1:1000, all raised in goat, all Invitrogen) in blocking buffer and Hoechst33258 (5 µg/mL) and mounted with Fluoromount^TM^ Aqueous Mounting Medium (Sigma-Aldrich, Burlington, MA, USA). For imaging, a Leica SP8 confocal microscope was utilized. 

The following primary antibodies were used: mouse anti-CB (1:500; Swant, Burgdorf, Switzerland), rat anti-CD31 (1:100; BD Biosciences Pharmingen, San Diego, CA, USA), rabbit anti-Cleaved Caspase-3 (Asp175) (1:250; Cell Signaling Technology, Danvers, MA, USA), mouse anti-CR (1:500; Swant, Burgdorf, Switzerland), guinea pig anti-Dcx (1:500; Merck Millipore, Burlington, MA, USA), rabbit anti-GABA (1:1000; Sigma-Aldrich, Burlington, MA, USA), chicken anti-Gfap (1:500; Labcorp Drug Development, Burlington, NC, USA), rabbit anti-Gfap (1:500; Sigma-Aldrich, Burlington, MA, USA), chicken anti-Gfp (1:500; Aves Labs, Tigard, OR, USA), rabbit anti-Iba1 (1:250; Abcam, Cambridge, UK), rabbit anti-Ki67 (1:250; Abcam, Cambridge, UK), mouse anti-NeuN (1:500; Merck Millipore, Burlington, MA, USA), rabbit anti-Olig2 (1:500; Atlas Antibodies, Bromma, Sweden), mouse anti-Satb2 (1:250; Abcam, Cambridge, UK), rat anti-Sox2 (1:500; eBioscience, Thermo Fischer Scientific, Waltham, MA, USA) and rabbit anti-Tbr2 (1:250; Abcam, Cambridge, UK).

### 2.4. Fluorescence In-Situ Hybridization (FISH)

Fresh-frozen cryo-sections containing mouse hippocampus were pre-treated as described before [21]. Coronal forebrain sections (20 µm) were defrosted and air-dried, followed by 1 h fixation in a 4% PFA in PBS solution (pH 7.4) and permeabilization in 0.4% Triton-X100 containing PBS for 10 min. Acetylation was carried out in 0.1 M tri-ethanolamine (pH 8.0) containing 0.25% (*v/v*) acetic anhydride. Sections were dehydrated and air-dried. Third-generation fluorescent ISH experiments (FISH) were performed as described elsewhere [22]. A probe against Oatp1c1 consisting of a set of 20 individual sequences for the target was commercially designed and generated (Molecular Instruments, Los Angeles, CA, USA). Sections were covered with hybridization buffer (Molecular Instruments, Los Angeles, CA, USA) for 10 min at 37 °C before a probe in the hybridization buffer (0.4 pmol in 100 µL) was applied and hybridization was performed for 24 h at 37 °C. Following rinsing with the probe washing buffer and 5× SSC + 0.1% Tween20 (SSCT), sections were incubated with the amplification buffer for 30 min at room temperature. Probe initiator-specific hairpins h1 and h2 labelled with AxF647 (6 pmol in 100 µL amplification buffer) were separately heat-shocked for 90 sec at 95 °C and cooled down at room temperature for 30 min. Hairpins were mixed in the amplification buffer and applied onto the slides. Signal amplification was performed for 16 h at room temperature. Next, slides were rinsed in SSCT, before immuno-fluorescence staining was carried out as above using mouse anti-NeuN (1:250; Merck Millipore, Burlington, MA, USA) and goat anti-mouse IgG-AxF488 (1:1000; Invitrogen, Thermo Fischer Scientific, Waltham, MA, USA) antibodies. Slides were briefly incubated with Hoechst33258 (5 µg/mL; 5 min), cover-slipped and imaged using a Leica SP8 confocal microscope. 

### 2.5. Open Field

Mice were placed in the middle of a 50 × 50 cm arena and exploratory behavior in an increasingly familiar environment was assessed for 30 min according to previously published protocols [23]. Analysis was performed using Viewer tracking software (Bonn, Germany). Tracks were analyzed for path length, visits and relative time spent in the central area (in-field, 15 × 15 cm), in the area close to the walls and in the corners, as well as walking speed, latency to move, time moving or resting and number of stops and rests.

### 2.6. Quantification

For FISH analysis, the number of Oatp1c1-mRNA/NeuN double positive cells was counted and calculated as a percentage of all NeuN immuno-positive cells. For immuno-histochemical analyses, marker positive cells (aCasp3, CB, CR, Dcx, EdU, Gfap, Ki67, Sox2, and Tbr2) in the SGZ were counted and normalized to the length of the SGZ using the open source program ImageJ (NIH) as described before [17]. Gfap+/Sox2+ NSC numbers were assessed in the middle of a broad z-stack, enabling the detection of a radial process extending into the granule cell layer. All quantifications were performed blindly by assigning random numbers to the animals. Five to six images from three to four sections per animal were subjected to quantification.

### 2.7. Statistics

All data represent mean + SEM. Two-way ANOVA followed by Bonferroni post-hoc testing was performed using GraphPad Prism 5. Differences were considered significant when *p* < 0.05 and were marked as follows: *, *p* < 0.05; **, *p* < 0.01; ***, *p* < 0.001. For behavioral analyses, statistical tests and parameter estimations were performed using SigmaStat 3.5 and SAS software 9.13 (SAS Institute Inc., Cary, NC, USA) and significance was assumed when *p* < 0.05. 

## 3. Results

### 3.1. Oatp1c1 Is Expressed in a Subset of Hippocampal Progenitor Cells and Mature Neurons

**Figure 1:**
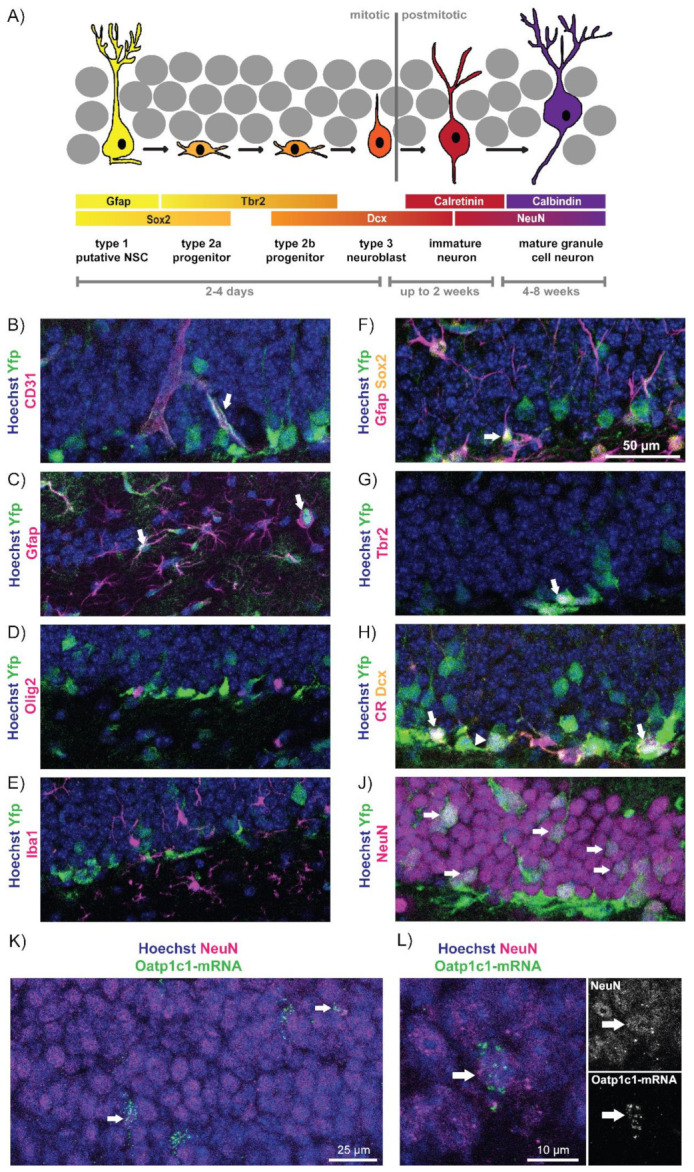
Oatp1c1 expression in the adult hippocampal lineage: (**A**) A schematic representation of the hippocampal neurogenic program, an illustration of stage-specific markers employed for immuno-fluorescence analyses as well as a timeline of lineage progression. At P60, male mice harboring the Oatp1c1-CreERT2 and an EYFP allele were injected for 5 days with tamoxifen and analyzed 3 days later. Yfp (in green) immuno-reactivity was found in (**B**) CD31 (magenta) positive endothelial cells and (**C**) Gfap (magenta) positive astrocytes, but not in (**D**) Olig2 (magenta) positive oligodendroglia cells and (**E**) Iba1 (magenta) positive microglia. In the neurogenic lineage, Yfp was detected in a subset of (**F**) NSCs positive for Gfap (magenta), Sox2 (yellow) and a radial process extending into the granule cell layer (arrow), (**G**) Tbr2 (magenta) positive TAPs, (**H**) Dcx+ (yellow), CR− (magenta) neuroblasts and Dcx+/CR+ immature neurons and (**J**) NeuN (magenta) positive neurons. The fresh-frozen cryo-sections obtained from P180 Wt males (n = 4) were subjected to combined detection of Oatp1c1-mRNA by FISH (in green) and NeuN protein (magenta) by immuno-fluorescence. The double positive cells are marked with an arrow. An overview (**K**) and a higher magnification (**L**) representative image are shown together with single Oatp1c1-mRNA and NeuN channels. The Hoechst33258 counter-stained nuclei are shown in blue.

To evaluate whether Oatp1c1 participates in the regulation of the neurogenic program in the adult hippocampus, we first examined if and in which cell types in the dentate gyrus this TH transporter is expressed. Adult hippocampal neurogenesis is a highly orchestrated program in which cells pass through different mitotic and post-mitotic stages that are distinguished by the expression of distinct marker proteins (Figure 1A) [24,25]. NSCs in the subgranular zone (SGZ) of the hippocampus positive for glial fibrillary acidic protein (Gfap) and SRY-box 2 (Sox2), and characterized by a radial process extending into the granule cell layer, divide asymmetrically in order to give rise to transiently amplifying progenitor cells (TAPs). These type 2 cells can be labelled with T-box brain protein 2 (Tbr2) and, after a short while, become neuroblasts (type 3 cells) positive for microtubule-associated protein doublecortin (Dcx). Neuroblasts then exit from the cell cycle and differentiate into immature neurons which up-regulate the Calcium-binding protein Calretinin (CR). The newly formed neurons slowly mature and integrate functionally into the existing granule cell network [26]. During this time, Dcx and CR expression ceases while Calbindin (CB) is up-regulated. While cells quickly pass through the mitotic stages within two to four days, postmitotic maturation and network integration is a slow process that can last between four to eight weeks after the cells have been born [27]. 

Due to the previously described Oatp1c1-specific antibody [18] no longer being available, we employed an inducible labeling approach by utilizing mice harboring a CreERT2 construct inserted into the endogenous Oatp1c1/Slco1c1 locus and an EYFP reporter allele. Following five consecutive days of tamoxifen treatment at the age of either two months (P60; Figure 1B–J, Figures S1A–E and S2) or six months (P180; Figures S1F–K and S3), mice were analyzed 72 h after the last injection.

At both time points, pronounced Yfp expression was visible in the dentate gyrus (Figure S1A and F, respectively). Here, Yfp co-localized with the endothelial marker CD31 (Figure 1B and Figure S1B,G) and the astrocyte marker Gfap (Figure 1C and Figure S1C,H), but was not found in oligodendroglia cells positive for Olig2 (Figure 1D and Figure S1D,J) nor in Iba1+ microglia (Figure 1E and Figure S1E,K). Within the neurogenic lineage, Yfp immuno-reactivity could be observed at P60 and P180 in NSCs positive for Gfap, Sox2 and extending a single process into the granule cell layer (Figure 1F, Figure S2A and Figure S3A, respectively), in Tbr2-positive TAPs (Figure 1G, Figure S2B and Figure S3B, respectively) as well as in Dcx+/CR- neuroblasts and Dcx+/CR+ immature neurons (Figure 1H, Figure S2C and Figure S3C, respectively). We detected Yfp immuno-reactivity in a subset of NeuN positive neurons at both time points (Figure 1J, Figure S2D and Figure S3D, respectively). These neurons do not co-localize with the GABAergic marker GABA (Figure S4A) and thus most likely represent granule cell neurons. This observation is not specific to the dentate gyrus, as we also detected Yfp positive signals in selected excitatory and non-neurogenic CA1 pyramidal cell neurons labelled by Satb2 (Figure S4B).

For validation, we performed combined FISH/immuno-fluorescence analysis on fresh-frozen brain sections obtained from six-month-old male Wt mice to detect endogenous Oatp1c1-mRNA in NeuN positive neurons in the dentate gyrus (Figure 1K,L). Oatp1c1 transcript signals were found scattered over the entire granule cell layer (Figure 1K) in both NeuN negative and NeuN positive cells (representative example of a double positive cell is shown in Figure 1L). Quantification revealed that 2.14 ± 0.14% of all NeuN-labelled neurons co-express Oatp1c1. Taken together, our results unequivocally demonstrate the presence of Oatp1c1 in a subset of progenitor cells and neurons in the dentate gyrus.

### 3.2. The Absence of Oatp1c1 Results in Distinct Impairments in Adult Hippocampal Neurogenesis

**Figure 2:**
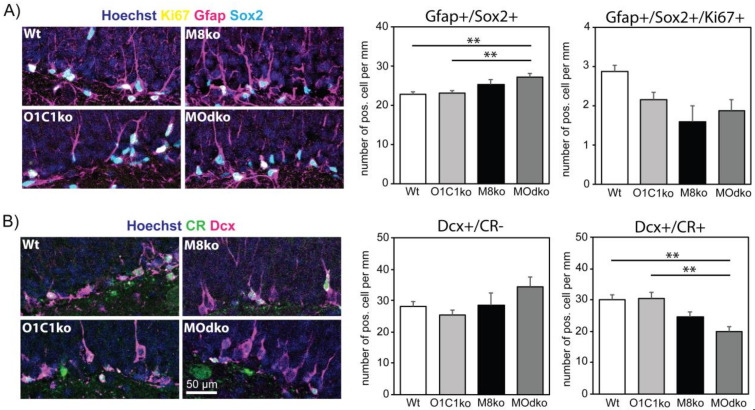
Impaired neurogenesis in two-month-old M/Odko mice: The perfusion-fixed coronal brain cryosections from two-month-old mice were subjected to immuno-histochemistry and the distinct neurogenic populations were analyzed and quantified. (**A**) NSCs were identified according to Gfap (magenta) and Sox2 (cyan) co-expression together with the presence of a radial process. NSCs positive for Ki67 (yellow) were regarded as active NSCs. (**B**) Dcx (magenta)/CR (green) co-labelling was used to distinguish type 2b progenitors and neuroblasts (Dcx+/CR−) from immature neurons (Dcx+/CR+). In all pictures, the Hoechst33258 counter-stained nuclei appear in blue. n = 6. Scale bar: 50 µm. **—*p* < 0.01. M8ko—Mct8ko; O1C1ko—Oatp1c1ko.

To investigate the impact of Oatp1c1 deficiency on the adult hippocampal neurogenic program and define the similarities and differences with the effects of Mct8 deficiency, we assessed the overall NSC numbers in two-month-old animals by Gfap/Sox2 co-labelling and enumerated those double positive cells that extend a radial process into the granule cell layer (Figure 2A). We examined four groups of animals: Wt controls, Oatp1c1ko and Mct8ko animals to examine the effect of the loss of each individual transporter in the neural cells, and M/Odko animals in which the loss of both transporters also results in a severely compromised TH uptake across the BBB and severe hypothyroidism throughout the CNS. No significant differences were detected between Wt and Oatp1c1ko animals in NSC numbers or proliferating, Ki67 positive NSCs (Gfap+/Sox2+/Ki67+). Equally, no significant differences were seen between the Oatp1c1ko animals and both the Mct8ko and the M/Odko animals. Likewise, other progenitor populations in the SGZ were unaffected in both sets of comparisons as no differences in Tbr2+ cells (Figure S5A) or neuroblasts (Dcx+/CR−) (Figure 2B) could been found. Similar numbers of apoptotic cells in the SGZ were found in all genotypes at P60 (Figure S5B). We conclude that, in two-month-old animals, neither Oatp1c1 nor Mct8 is required in the neural cells for the early stages of hippocampal neurogenesis. At later stages, and as we have previously described, Mct8 plays a role in neurogenesis as the density of immature neurons (Dcx+/CR+; Figure 2B) was significantly reduced to a similar extent both in Mct8ko and M/Odko mice. At this later stage, however, Oatp1c1 is still not required in these two-month-old animals as there were no differences between Wt and Oatp1c1ko animals (Figure 2B).

**Figure 3:**
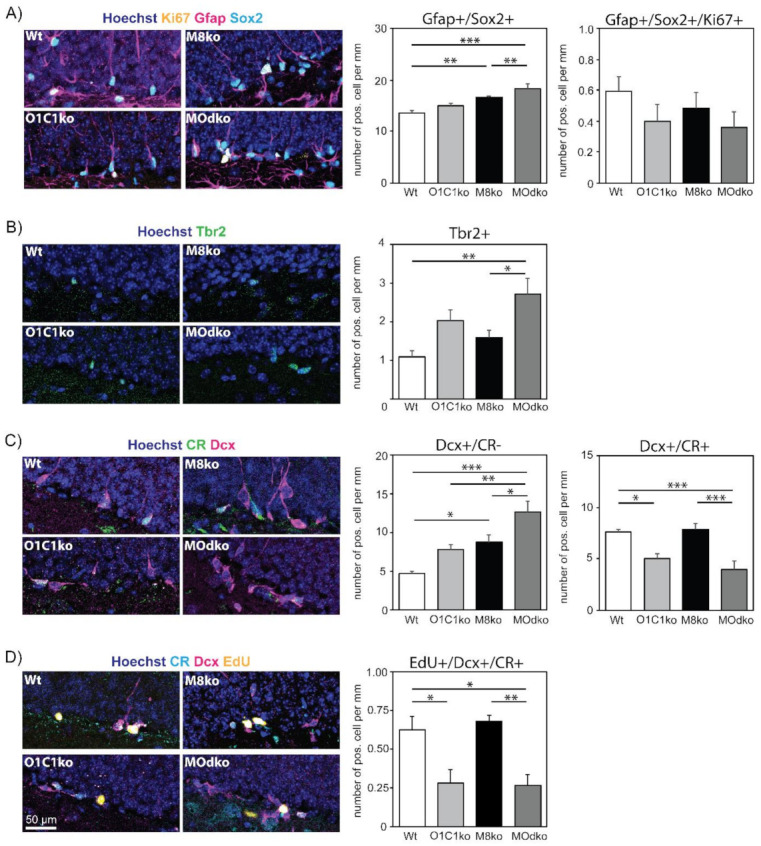
Altered hippocampal neurogenesis in six-month-old Oatp1c1 ko and M/O dko mice: Neurogenic stages were analyzed at six months of age. (**A**) Total NSC numbers (Gfap+ (magenta), Sox2+ (cyan) and extending a radial process) as well as the Ki67+ (yellow) activated NSCs were assessed. (**B**) The number of type 2 progenitors was assessed based on Tbr2 (in green) expression. (**C**) The Dcx (magenta) only positive neuroblasts (Dcx+/CR−) and the Dcx/CR (green) double positive immature neurons (Dcx+/CR+) were visualized and quantified. (**D**) EdU (yellow) incorporation into Dcx+ (magenta)/CR+ (cyan) double positive immature neurons was assessed three days after label injection. The cell nuclei were stained with Hoechst33258 (blue) in all experiments. n = 6. Scale bar: 50 µm. *—*p* < 0.05; **—*p* < 0.01; ***—*p* < 0.001. M8ko–Mct8ko; O1C1ko–Oatp1c1ko.

We next repeated our analyses in 6-month-old animals, an age at which the efficiency of the hippocampal neurogenic program is compromised [28,29]. Again, no significant differences were detected between Wt and Oatp1c1ko animals in NSC numbers. Due to a non-cell autonomous effect of Mct8 loss [17], Mct8ko and M/Odko mice displayed significantly elevated and similar numbers of Gfap/Sox2 double positive NSCs extending a radial process (Figure 3A; Gfap+/Sox2+). NSC activation as determined by Ki67 immuno-positivity tended to be decreased in all knockout groups (Gfap+/Sox2+/Ki67+), but these results were not statistically significant. M/Odko animals exhibited an increased density of Tbr2+ transiently amplifying progenitors (Figure 3B). Examining later stages of the neurogenic lineage, Oatp1c1ko animals showed increased numbers of Dcx+/CR− neuroblasts in the SGZ, with a similar increase also seen in the Mct8ko animals and an even higher density found in M/Odko mice (Figure 3C). As before, the number of aCasp3+ apoptotic cells was not significantly different between the genotypes (Figure S5C). Post-mitotic immature neurons (Dcx+/CR+) were reduced in the Oatp1c1ko animals as compared to Wt. There were no significant changes in the Mct8ko mice (Figure 3C), despite the increase in the Dcx+/CR− neuroblasts that represent the preceding step in the differentiation pathway. M/Odko mice showed a reduction in Dcx+/CR+ cells similar to that seen in the Oatp1c1ko animals. This indicates that both TH transporters contribute to the TH-dependent regulation of the adult hippocampal neurogenic program at the transition from Dcx+/CR− neuroblast to Dcx+/CR+ immature neurons in these older animals. The weak effect of Mct8 is evidenced by the discrepancy between the increased pool of Dcx+/CR− neuroblasts and normal numbers of Dcx+/CR+ immature neurons. A stronger effect of Oatp1c1 at this step is revealed by the finding that, in the absence of this transporter, the number of Dcx+/CR+ immature neurons is reduced even in the presence of an increased pool size of Dcx+/CR− cells.

To substantiate this finding, we analyzed EdU incorporation into immature neurons in P180 animals three days after label injection (Figure 3D and Figure S5D). The absence of Oatp1c1 compromised the formation of new, EdU+ immature neurons, which were reduced in number to a similar extent in both Oatp1c1ko and M/Odko mice. These results confirm a role of Oatp1c1 in the neurogenic program in these older mice.

**Figure 4:**
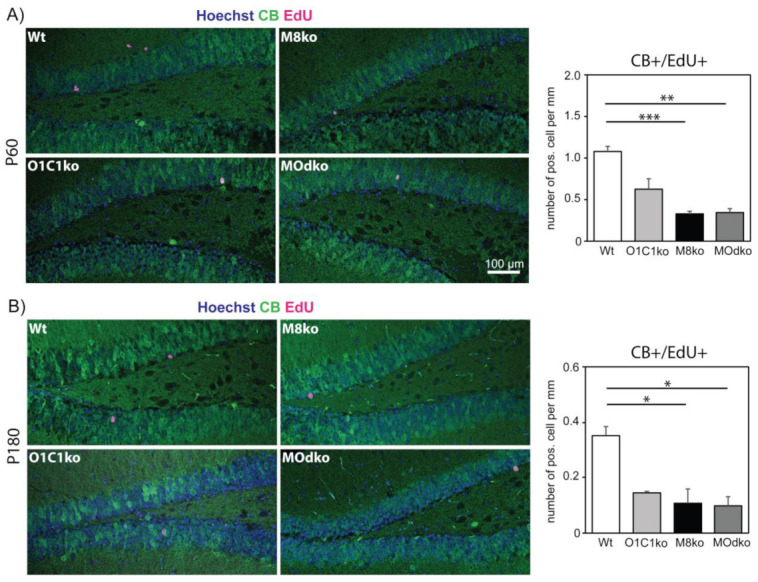
Granule cell neuron generation is impaired in TH transporter deficiency: A single EdU pulse was injected at P60 (**A**) or P180 (**B**). 28 days after injection, EdU (magenta) retention in mature granule cell neurons (CB+, green) was analyzed. The nuclei (blue) were stained by Hoechst33258. n = 4. Scale bar: 100 µm. *— *p* < 0.05; **—*p* < 0.01; ***—*p* < 0.001. M8ko—Mct8ko; O1C1ko—Oatp1c1ko.

Together, these results show that Mct8 and Oatp1c1 are required for progression to the Dcx+/CR+ immature neuron stage. To determine whether loss of these transporters then reduces the capacity to form and integrate new granule cell neurons, the final stage of hippocampal neurogenesis, we employed EdU pulse-chase experiments and quantified the number of new, CB+/EdU+ neurons 28 days after a single EdU pulse was injected at P60 or P180 (i.e., in both young and old animals; Figure 4A and B, respectively). A reduction in new granule neurons was seen in all the different ko mice at both ages, although the effect was greater (and only achieved statistical significance) in the absence of Mct8 alone or in combination with Oatp1c1. These results suggest that the capacity to generate new granule cell neurons depends more on Mct8 than Oatp1c1.

### 3.3. The Absence of Mct8 and Oatp1c1 Reduces Exploratory Behavior

**Figure 5:**
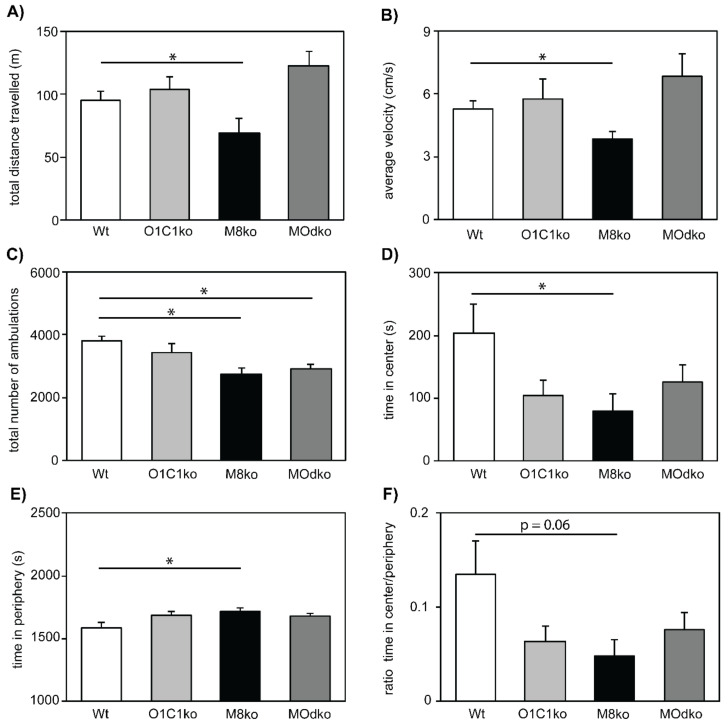
TH transporter deficiency results in behavioral abnormalities in the open field arena: Female mice at the age of one year were tracked for 30 min in an open field arena. The following parameters are displayed: (**A**) The total distance the mice travelled within 30 min, (**B**) their average velocity over the entire time, (**C**) the total number of ambulations over all areas, (**D**) the total time spent in the center squares, (**E**) the total time spent in the periphery, and (**F**) the ratio of time in the center/periphery. n = 7. *—*p* < 0.05; M8ko—Mct8ko; O1C1ko—Oatp1c1ko.

To address if deficits in adult hippocampal neurogenesis seen with the loss of either transporter in the older animals result in an altered hippocampus-related behavioral performance, we analyzed exploration activity and anxiety in one-year-old mice using an open field arena (Figure 5). Total track length and average velocity were significantly reduced only in M8ko mice (Figure 5A,B, respectively). Mct8-deficient groups presented a lower overall number of ambulations, indicating reduced exploratory behavior, reduced curiosity and probing (Figure 5C). In agreement with these findings, while all groups showed a strong tendency to avoid the inner zone and thus signs of increased anxiety (Figure 5D), statistically significant p values were found for Mct8ko mice only. Likewise, while all knockout groups tended to remain in peripheral squares, statistically significant changes were detected for Mct8ko mice only (Figure 5E). The strong tendency of all TH transporter mouse mutants to avoid the central squares is also illustrated in Figure 5F, where the ratio of time spent in the center/periphery is depicted. Other analyzed parameters were not different between the genotypes. We conclude that, while the effects of Mct8 loss cause significant differences in anxiety-related behavior, the loss of Oatp1c1 has no significant effect. This likely reflects the finding above that the reduction in new granule cells neurons in the presence of Oatp1c1 loss was not significant, while that in the presence of Mct8 loss was significant.

## 4. Discussion

A growing body of evidence highlights that in adult hippocampal neurogenesis, TH controls the transition from proliferating neuroblasts to post-mitotic immature neurons while also stimulating the survival of later-stage mitotic and post-mitotic cells [3,30,31]. In order to fulfill its actions, TH has to be taken up into target cells—a process for which TH transporters are mandatory [4]. Using global and conditional knockout mouse models of Mct8, the most specific TH transporter known so far, we have recently identified a cell-autonomous function of Mct8 in the neurogenic lineage for the differentiation of adult-born neuroblasts to immature and mature neurons as well as indications of non-cell-autonomous effects in niche cells [17]. Here, we addressed the question of whether the TH transporter Oatp1c1 is also present within the hippocampal neurogenic lineage and stem cell niche and whether it acts alone or in concert with Mct8 in regulating hippocampal neurogenesis. Our results confirm Oatp1c1 expression in the lineage and demonstrate a role in the transition from Dcx+/CR− neuroblasts to Dcx+/CR+ immature neurons in older animals within which the overall rate of neurogenesis has declined from levels seen in younger animals.

To examine Oatp1c1 expression, we used an Oatp1c1-CreERT2-Yfp reporter mouse. These animals report Oatp1c1 promoter activity by assessing Yfp immuno-reactivity that is induced upon tamoxifen application. At two analyzed time points (two-month- and six-month-old animals), Yfp immuno-reactivity could be detected in subsets of neural stem cells, type 2 progenitor cells, neuroblasts and both immature and mature granule cells within the dentate gyrus SGZ. These findings align with previous reports, using the same tamoxifen-inducible Oatp1c1-CreERT2 or a constitutively active Oatp1c1-Cre mouse model, where a subset of dentate gyrus granule cells were shown to be positive for the tdTomato reporter [19,32,33]. We cannot exclude that the Yfp immuno-reactivity in these distinct cell types within the neurogenic lineage may result from their formation by NSCs with an activated Yfp reporter that have generated daughter cells which have progressed within the neurogenic program since the first tamoxifen injection. However, Oatp1c1-specific FISH signals in NeuN positive granule cells provide strong evidence for an endogenous expression of Oatp1c1 in a subset of mature granule neurons. Whether this subset-specific Oatp1c1 expression represents a temporary phase in the life of granule neurons (e.g., early after they have acquired maturity) or whether this points to granule neuron heterogeneity [34,35] remains to be determined.

For elucidating the cellular function of Oatp1c1 within the neurogenic niche, we analyzed Oatp1c1ko mice and compared our results with recent observations obtained in Mct8ko animals as well as Mct8/Oatp1c1 dko animals. Oatp1c1 deficiency is characterized by an accumulation of Dcx+/CR− cells in the SGZ (Figure 3C) together with reduced neuroblast differentiation capacity, although this phenotype becomes apparent only in six-month-old animals. These findings demonstrate a function of Oatp1c1 in governing the neuroblast-to-immature neuron transition. The fate of these accumulated Dcx+/CR− cells is presently unknown. Though we failed to detect any change in the number of apoptotic cells, we currently cannot exclude that necrosis or autophagic cell death is altered in Oatp1c1 deficiency [36]. In comparison to Oatp1c1, Mct8 appears to play a role in both the earlier transition to immature neurons and the survival, migration and/or functional integration of newborn neurons in the last step of the neurogenic program, as the number of newborn neurons is more strongly decreased in Mct8ko and M/Odko animals compared to Oatp1c1ko mice. We conclude that each transporter plays a necessary role at different stages of the neurogenic process, perhaps explaining in part the different human phenotypes that result from their loss due to genetic mutations.

It remains to be investigated to what extent the alterations seen in Oatp1c1 deficient mice can be attributed to the lack of Oatp1c1 in NSCs and their progeny or to what extent overall alterations in the thyroidal state of the CNS compromise the differentiation of neuroblasts—in other words, whether the effect is cell-autonomous or non-autonomous. This needs to be investigated by the use of conditional knockout models lacking Oatp1c1 only in the hippocampal lineage. Stem cell niche cells may represent sensors and mediators of any non-cell-autonomous effects. We could detect Yfp immuno-reactivity in some astrocytes and the majority of endothelial cells in agreement with previous observations [19,32]. Of note, both cell types are important constituents of the hippocampal stem cell niche [37]. Oatp1c1 in endothelial cells contributes significantly to the T4 transport across the BBB as Oatp1c1ko mice exhibit a slightly reduced brain T4 content while brain T3 concentration and T3 signaling are rather normal [18]. Mct8 in the same cell type plays a pivotal role in facilitating T3 access to the CNS [10]. Consequently, the concomitant inactivation of both TH transporters Mct8 and Oatp1c1 in mice results in a strongly diminished uptake of T3 and T4, and thus a profound TH deficient state in the CNS [12]. It is therefore feasible that, in addition to any cell autonomous effects, an impaired TH transport in BBB endothelial cells due to a lack of either or both Oatp1c1 and Mct8 indirectly compromises hippocampal neurogenesis, particularly as endothelial cell-derived brain derived neurotrophic factor (Bdnf) or vascular endothelial growth factor (Vegf) that stimulate neurogenesis are sensitive to TH [18,37,38]. Thus, in the future, it will be important to address the impact of the TH transporter Oatp1c1 in these niche cells on the adult hippocampal neurogenic program and on these TH regulated genes by conditional knockout strategies. Such conditional knockout mice would also help to clarify why Oatp1c1ko mice display an altered neurogenesis only at six months of age and to what extent changes in astrocytes and/or endothelial cell homeostasis contribute to this phenotype.

A collaborative role of Mct8 and Oatp1c1 has recently been demonstrated in the second well-established neurogenic area in the adult CNS, the subventricular zone [39]. Here, their combined absence impaired the proliferation of progenitor cells and was further linked to an impaired olfactory memory, thus also highlighting a role of TH transporters for adult neurogenesis in vivo. Moreover, our observations that Mct8ko mice display anxiety-related behavioral abnormalities, despite unaltered locomotor activity [12], are in agreement with prior studies on mice globally expressing a mutant form of the thyroid hormone receptor alpha 1 (TRa1) with a 10x lower affinity towards TH. Those TRa1 mutant mice demonstrated increased anxiety in the elevated plus maze test and reduced exploratory behavior in the open field arena [40]. More recently, increased anxiety was also observed in mice with astrocyte-specific ablation of the T4-to-T3-converting deiodinase type 2 [41]. Mood disorders such as anxiety, hippocampus-related memory defects and a reduced hippocampal volume are hallmarks of adult-onset hypothyroidism in patients. In concert, our results and those from other studies suggest that these symptoms may in part be explained by impaired adult hippocampal neurogenesis [42,43,44,45].

Together, our findings advance the idea that proper TH transport across brain barriers and into neural cells is essential for the maintenance of adult brain functions such as adult hippocampal neurogenesis. We establish Oatp1c1 as an important regulator of neuroblast-to-immature neuron transition in the adult SGZ. Impairments in this transitory process may potentially explain some of the neurodegenerative aspects that have been observed in a human OATP1C1 patient. In this respect, our study will inform therapeutic approaches and long-term treatment strategies for patients suffering from OATP1C1 deficiency.

## Supplementary Materials

The following are available online at https://www.mdpi.com/article/10.3390/cells11030524/s1, Figure S1: Oatp1c1 expression in non-neuronal cells, Figure S2: Oatp1c1 expression in adult hippocampal lineage cells at P60, Figure S3: Oatp1c1 expression in adult hippocampal lineage cells at P180, Figure S4: Oatp1c1 expression in neuronal subtypes, Figure S5: Progenitors in the SGZ in TH transporter deficiency.
