# Supplementary material for: Distinct Actions of the Thyroid Hormone Transporters Mct8 and Oatp1c1 in Murine Adult Hippocampal Neurogenesis
Source: Cells. 2022 Feb 2;11(3):524. doi: 10.3390/cells11030524 (PMC8834272; doi:10.3390/cells11030524)
Supplement: Supplementary file 1 [file cells-11-00524-s001.zip › cells-1504563-supplementary.pdf]

cells

MDPI

Supplementary Materials

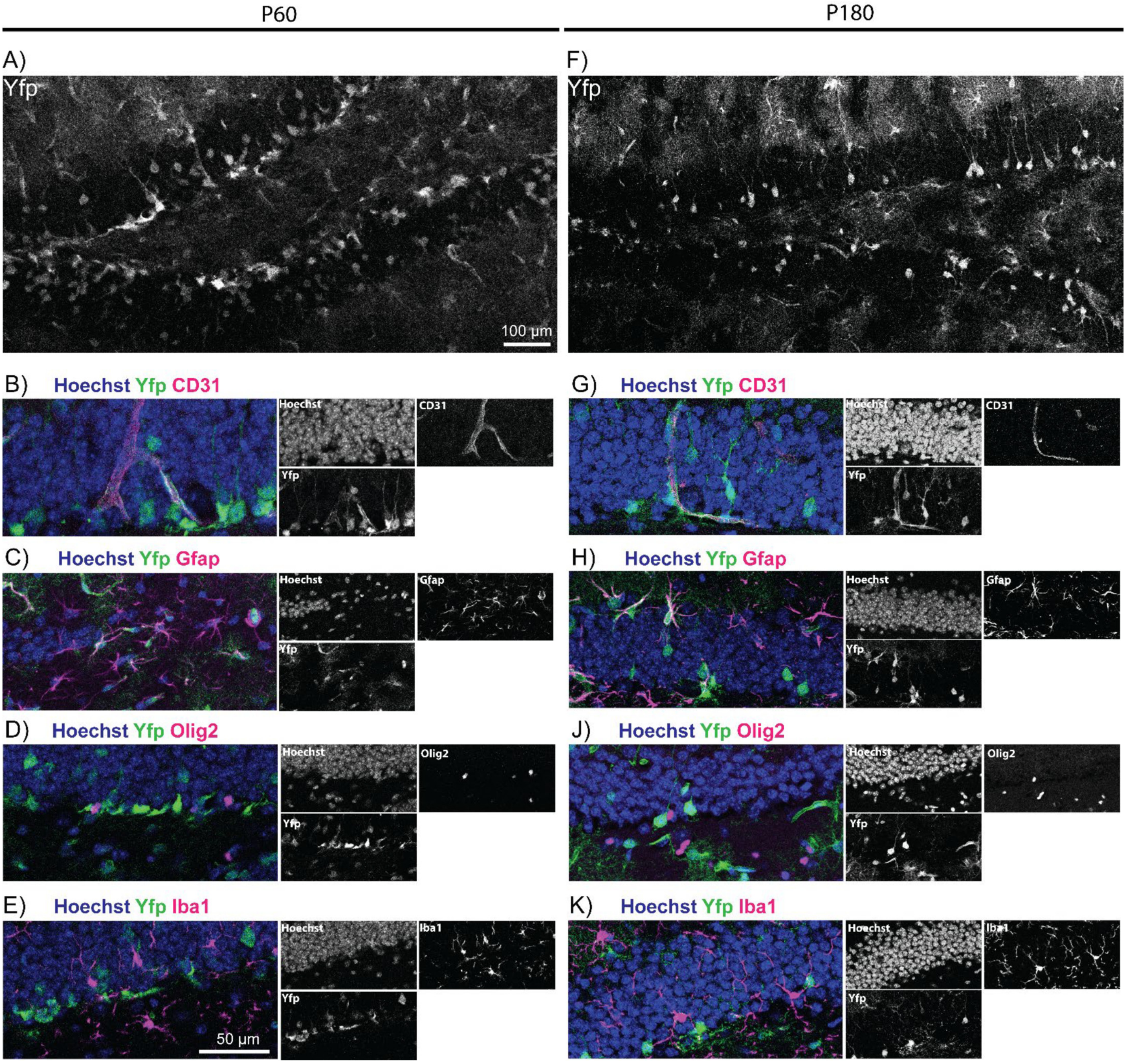

Figure S1. Oatp1c1 expression in non-neuronal cells: Male mice carrying the Oatp1c1-CreERT2 and an EYFP allele were subjected to 5 days of tamoxifen treatment and analysed 72 h after the last injection. (**A**) Using P60 mice, pronounced Yfp expression was seen in the dentate gyrus area. In detail, Yfp (in green) immuno-reactivity was visible in subsets of (**B**) CD31 (magenta) positive endothelial cells, (**C**) Gfap (magenta) positive astrocytes, but not in (**D**) Olig2 (magenta) positive oligodendroglia cells and (**E**) Iba1 (magenta) positive microglia. Likewise, mice were subjected to the same treatment at the age of 6 months (P180). Again, Yfp (in green) positive signals were observed in the entire dentate gyrus area (**F**) and specifically in a subset of (**G**) CD31 (magenta) positive endothelial cells, (**H**) Gfap (magenta) positive astrocytes, but not in (**J**) Olig2 (magenta) positive oligodendroglia cells and (**K**) Iba1 (magenta) positive microglia. Single channel pictures are presented in grey scale. Scale bars: 100 µm (A and F), 50 µm (B-E and G-K).

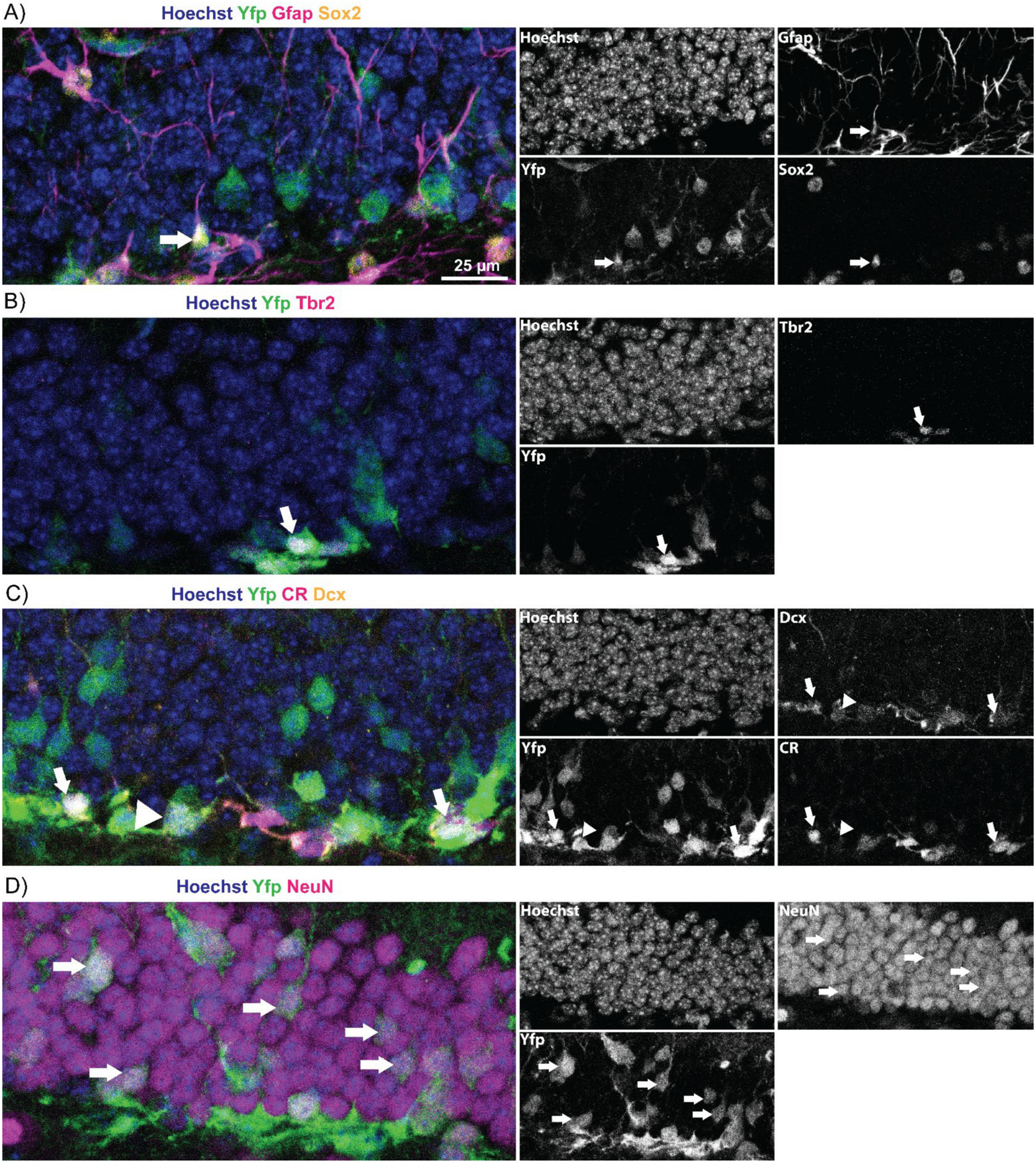

Figure S2. Oatp1c1 expression in adult hippocampal lineage cells at P60. Yfp (green) immuno-reactivity was detected in (**A**) Gfap+ (magenta), Sox+2 (yellow) NSCs that extend a radial process into the granule cell layer (arrow), (**B**) Tbr2+ (magenta) TAPs; (**C**) Dcx+ (yellow), CR- (magenta) neuroblasts and Dcx+/CR+ immature neurons; and (**D**) NeuN+ (magenta) neurons. Hoechst33258 labelled nuclei appear blue. Single channel pictures are displayed in grey scale. Scale bar: 25 µm.

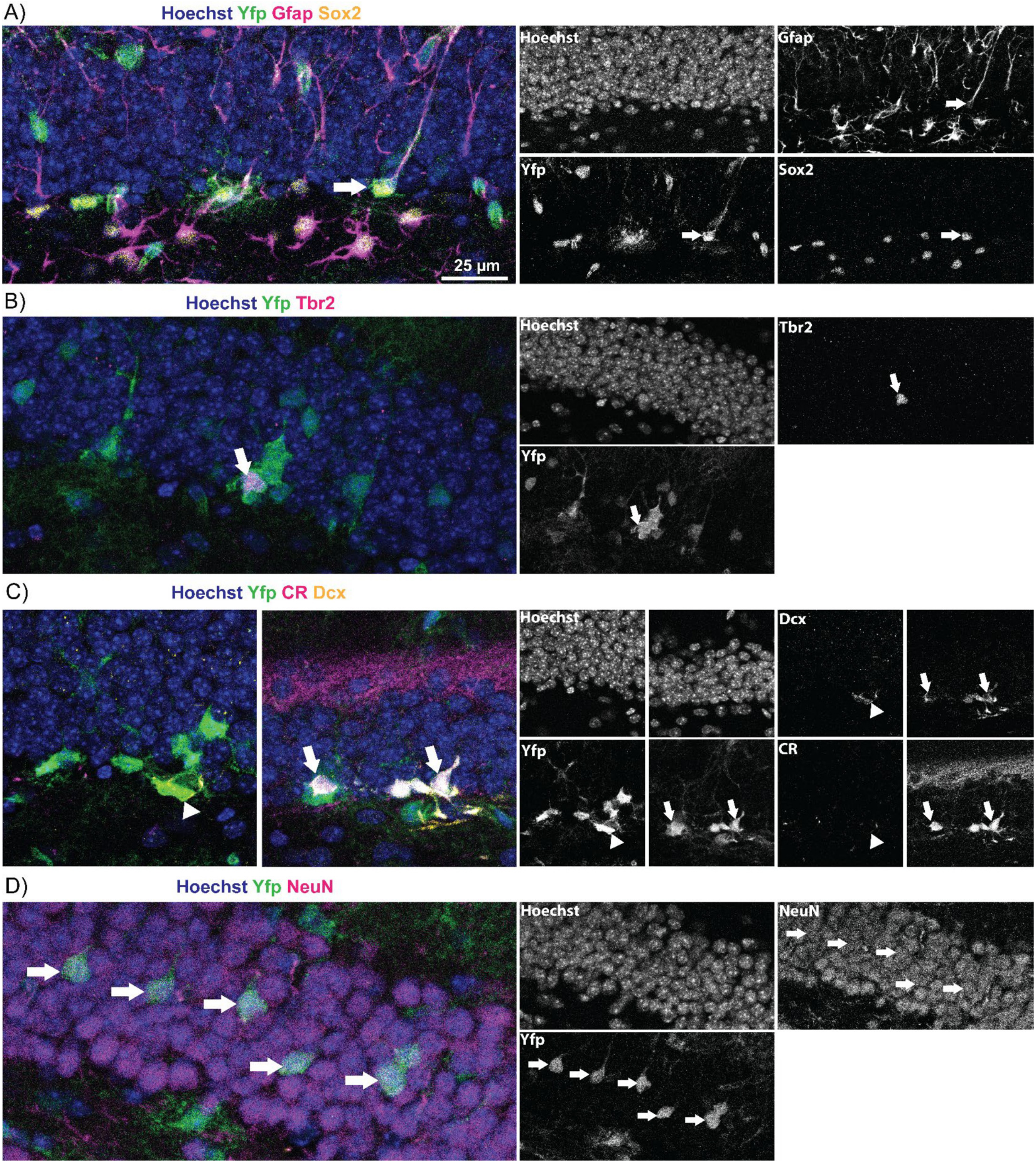

Figure S3. Oatp1c1 expression in adult hippocampal lineage cells at P180. Yfp immuno-reactivity shown in green was present in subsets of (**A**) Gfap+ (magenta), Sox+2 (yellow) NSCs extending a single radial process into the granule cell layer (arrow), (**B**) Tbr2+ (magenta) TAPs; (**C**) Dcx+ (yellow), CR- (magenta) neuroblasts and Dcx+/CR+ immature neurons, and (**D**) NeuN+ (magenta) neurons. Cell nuclei were counter-stained with Hoechst33258 (blue). Single channel pictures are presented in grey scale. Scale bar: 25 µm.

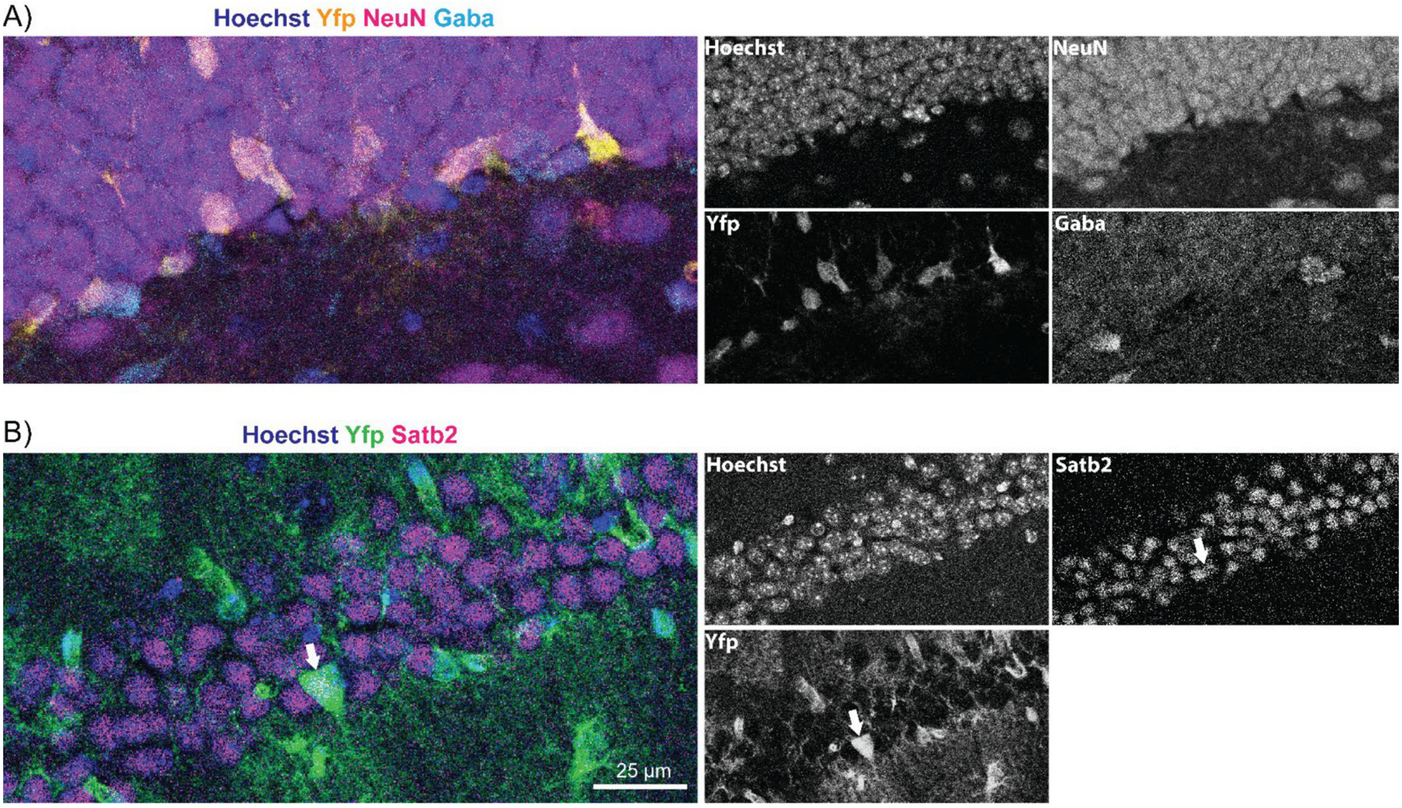

Figure S4. Oatp1c1 expression in neuronal subtypes. (**A**) Yfp (yellow)/NeuN (magenta) double positive neurons in the granule cell layer of P180 animals do not co-localize with GABA (cyan). (**B**) In the CA1 area of P60 animals, distinct pyramidal cell neurons positive for Satb2 (magenta) co-express Yfp (green). In both co-labelling studies, Hoechst33258 counter-stained nuclei are depicted in blue. Single channel pictures are shown in grey scale. Scale bar: 25 µm.

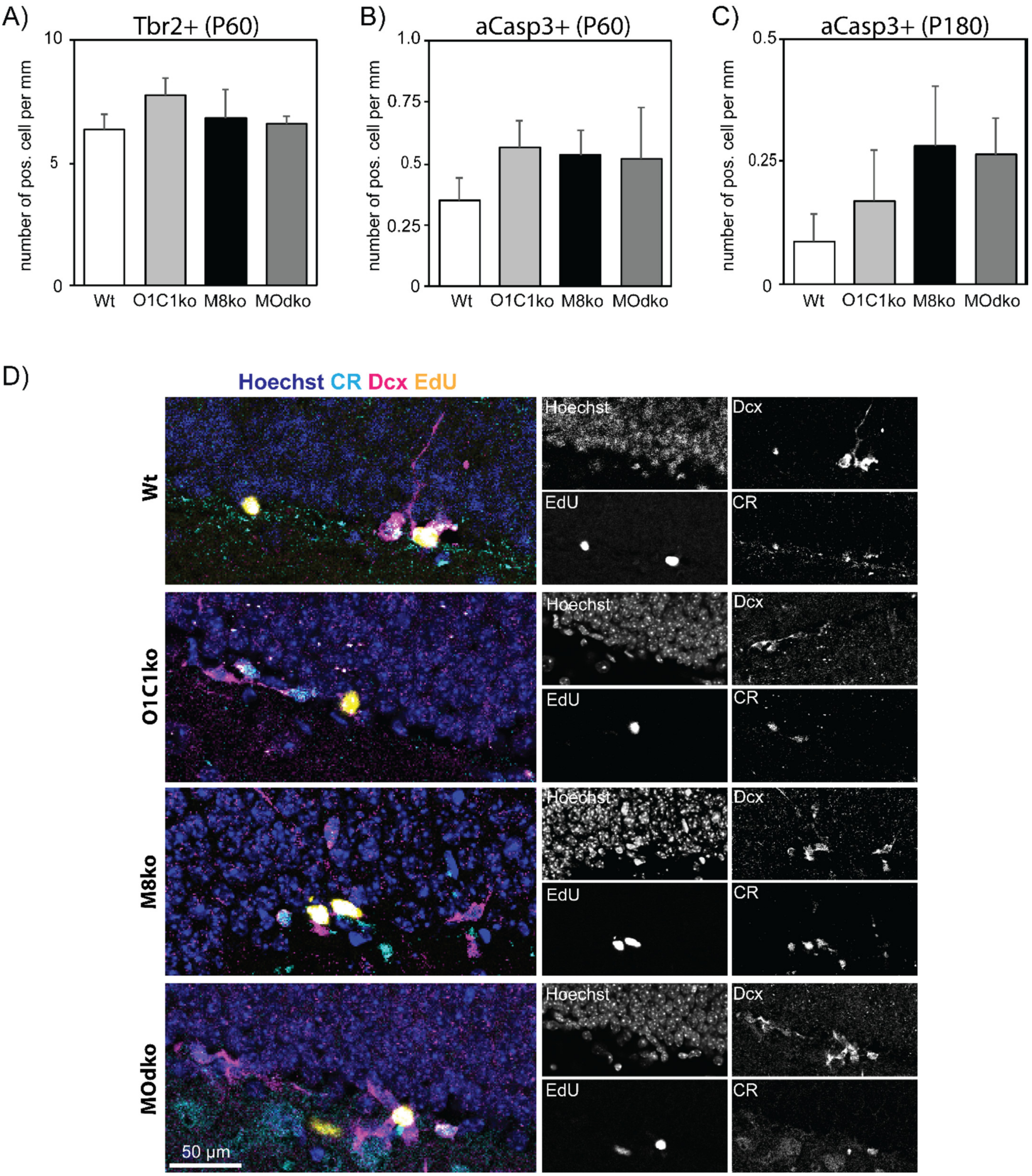

Figure S5. Progenitors in the SGZ in TH transporter deficiency. Transiently amplifying progenitors were analysed and numbers of Tbr2 positive cells were enumerated at P60 (**A**). Cleaved caspase 3 (aCasp3) positive cell numbers were quantified at P60 (**B**) and P180 (**C**). (**D**) 3 days after label injection, EdU (yellow) incorporation into Dcx+ (magenta) /CR+ (cyan) double positive immature neurons was analysed at P180. Hoechst33258 labelled cell nuclei are shown in blue. Single channel pictures are displayed in grey scale. Scale bar: 50 µm.
